# Impact of isolation methods on the biophysical heterogeneity of single extracellular vesicles

**DOI:** 10.1038/s41598-020-70245-1

**Published:** 2020-08-07

**Authors:** Shivani Sharma, Michael LeClaire, James Wohlschlegel, James Gimzewski

**Affiliations:** 1 Department of Pathology & Laboratory Medicine, David Geffen School of Medicine, University of California Los Angeles, Los Angeles, 90095 USA; 2 California NanoSystems Institute, University of California Los Angeles, Los Angeles, CA 90095 USA; 3 Jonsson Comprehensive Cancer Center, University of California Los Angeles, Los Angeles, CA 90095 USA; 4 Department of Chemistry & Biochemistry, University of California Los Angeles, Los Angeles, CA 90095 USA; 5 Department of Biological Chemistry, University of California Los Angeles, Los Angeles, CA 90095 USA

**Keywords:** Nanoscale biophysics, Atomic force microscopy, Scanning probe microscopy, Super-resolution microscopy, Characterization and analytical techniques, Breast cancer, Biological techniques, Biophysics, Cancer, Structural biology, Nanoscience and technology

## Abstract

Extracellular vesicles (EVs) have raised high expectations as a novel class of diagnostics and therapeutics. However, variabilities in EV isolation methods and the unresolved structural complexity of these biological-nanoparticles (sub-100 nm) necessitate rigorous biophysical characterization of single EVs. Here, using atomic force microscopy (AFM) in conjunction with direct stochastic optical reconstruction microscopy (dSTORM), micro-fluidic resistive pore sizing (MRPS), and multi-angle light scattering (MALS) techniques, we compared the size, structure and unique surface properties of breast cancer cell-derived small EVs (sEV) obtained using four different isolation methods. AFM and dSTORM particle size distributions showed coherent unimodal and bimodal particle size populations isolated via centrifugation and immune-affinity methods respectively. More importantly, AFM imaging revealed striking differences in sEV nanoscale morphology, surface nano-roughness, and relative abundance of non-vesicles among different isolation methods. Precipitation-based isolation method exhibited the highest particle counts, yet nanoscale imaging revealed the additional presence of aggregates and polymeric residues. Together, our findings demonstrate the significance of orthogonal label-free surface characteristics of single sEVs, not discernable via conventional particle sizing and counts alone. Quantifying key nanoscale structural characteristics of sEVs, collectively termed ‘EV-nano-metrics’ enhances the understanding of the complexity and heterogeneity of sEV isolates, with broad implications for EV-analyte based research and clinical use.

## Introduction

The emerging roles of secreted nanometer-sized exosomes or other extracellular vesicles (EVs)^1^, in various physiological and pathological processes, has led to enormous interest in their potential clinical utility as cell-free biomarkers for cancer and other diseases^2^. Consequently, numerous efforts are underway to isolate, characterize, and optimize EVs^3–6^. However critical knowledge gaps still exist in our understanding of the biophysical properties of single EVs. There is no single “gold standard” method used for the isolation of EVs. Further, the current biophysical analyses of EVs typically encompass inadequate particle counting and size distribution determinations using various techniques. Together, to overcome the barriers due to the structural complexity (heterogeneity, small-to-large size ~ 30–1000 nm) of these biological-nanoparticles, and isolation method dependent variabilities, improved biophysical analysis of EV isolates that complement and inform existing biomolecular analysis techniques is warranted prior to their subsequent use for reliable biomarkers or as therapeutics.

The small EV (sEV) isolates from commonly used methods such as ultracentrifugation (UC)^7^, density ultracentrifugation (UCg)^8^, size exclusion^9^ or immuno-affinity (IA)^7,10^ not only vary in purity and yield, but additionally demonstrate structural and biomolecular heterogeneity^11,12^. Further, precipitation (PT) based approaches represent an easy and fast approach for EV isolation which is mostly exploited by commercial kits. Several biophysical techniques estimate the particle counts and size distributions^13–17^, including nano-flow cytometry^6,18^, resistive pulse sensing^19^, nanoparticle tracking analysis^20^, and micro-fluidic approaches^11,21–24^. Yet, uncertainties and challenges exist with respect to reliable EV enumeration and comparisons of EV isolates obtained using different isolation techniques. Notably, most particle characterization approaches for evaluating EV isolates rely heavily upon particle size and count determinations but invariably fail to capture additional high-resolution characteristics such as structure, surface topography, adhesiveness or elasticity of single EVs which may offer novel orthogonal markers to precisely quantify the EV biophysics and nanoscale effects of isolation strategies used.

Structurally, electron microscopy (EM)^25^ has provided a wealth of information on sEVs^12^ given their nanoscale size range^5,6,26^, and while EM gives qualitative 3D information, quantitative computations on particle surfaces are challenging. Advancements in optical super-resolution imaging approaches further bring new capabilities to visualization of labeled sEVs. By contrast, atomic force microscopy (AFM)^27^ enables deriving label-free, 3D, quantitative information of sEV isolates at sub-nanometer scale resolution^28^. Nevertheless, only a limited range of biophysical characteristics have been explored to date to assess the nanoscale scale impact of isolation methods on EV isolates. Here, using atomic force microscopy (AFM) in conjunction with direct stochastic optical reconstruction microscopy (dSTORM), micro-fluidic resistive pore sizing (MRPS), and multi-angle light scattering (MALS) techniques, we compared the size, structure and unique surface properties of breast cancer cell-derived sEVs obtained using different isolation methods. We identify novel biophysical properties to benchmark both the biophysical “quantity and quality” of EV isolates that largely remain obscured in the ensemble or other characterization techniques. Our findings reveal that the quantification of key biophysical parameters within sEV isolates collectively termed ‘EV-nano-metrics’ provides novel orthogonal markers to precisely quantify the nanoscale effects of isolation strategies at the single particle level. The findings hold significant potential implications for EV-based downstream applications particularly where molecular markers of sEVs are not established or not available.

## Results

**Figure 1:**
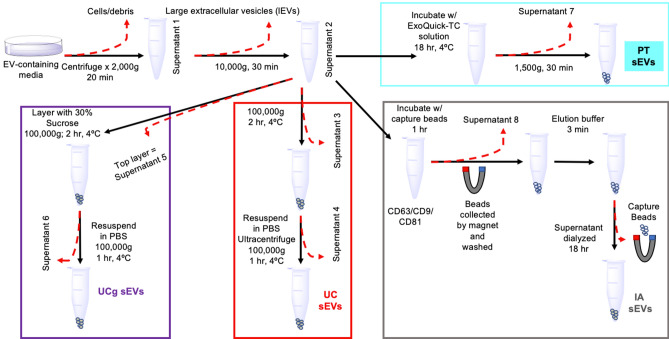
Protocol used for simultaneous isolation of sEVs from breast cancer cells (MCF7, MDA-MB-231) using four different isolation methods (i.e., ultracentrifugation- UC, sucrose density ultracentrifugation- UCg, Immune Affinity-IA, and polymeric precipitation-PT).

To quantify the nanoscale variations in sEV isolates obtained using different isolation methods (Fig. 1) and cell types, key biophysical sEV-nano-metrics (namely particle morphology, counts, size distributions, surface roughness) were derived using multiple techniques (as summarized in Table 1).

**Table 1:** Biophysical characteristics of sEV particles obtained from breast cancer cells, using different isolation methods.

Isolation method	Cell line	AFM						dSTORM		MALS		MRPS	
		Particle counts	Mean ± SD particle diameter (nm)		Average particle Rms surface roughness (nm)			Mean ± SD particle diameter (nm)		Median particle diameter (nm)		Median particle diameter (nm)	
			*Pk 1*	*Pk 2*	*Mean*	*SD*	*variance*	*Pk 1*	*Pk 2*	*Pk 1*	*Pk 2*	*Pk 1*	*Pk 2*
UC	MDA-MB-231	727	69.3 ± 18	–	0.23	0.03	0.001	74 ± 22	–	46.6	–	< detection	–
	MCF7	724	62.2 ± 6	–	0.26	0.04	0.001	70 ± 16	–				
UCg	MDA-MB-231	174	70.5 ± 18.9	–	0.42	0.01	3.8 × 10^−4^	70 ± 20	–	120.6	–	–	–
	MCF7	163	71.0 ± 16	–	0.48	0.01	2.2 × 10^−4^	76 ± 12	–				
IA	MDA-MB-231	628	82.0 ± 38	140 ± 8	0.71	0.09	0.008	54 ± 12	79 ± 10	99.7	213.4	65	–
	MCF7	505	75.5 ± 22	135 ± 18	0.59	0.04	0.001	48 ± 12	86 ± 8				
PT	MDA-MB-231	1,190	88.4 ± 36.4	–	3.5	0.37	0.13	na	na	99	210		
	MCF7	1515	100.4 ± 73.0	–	2.83	0.55	0.30						

### Comparative AFM based structural characterization of sEV isolates

**Figure 2:**
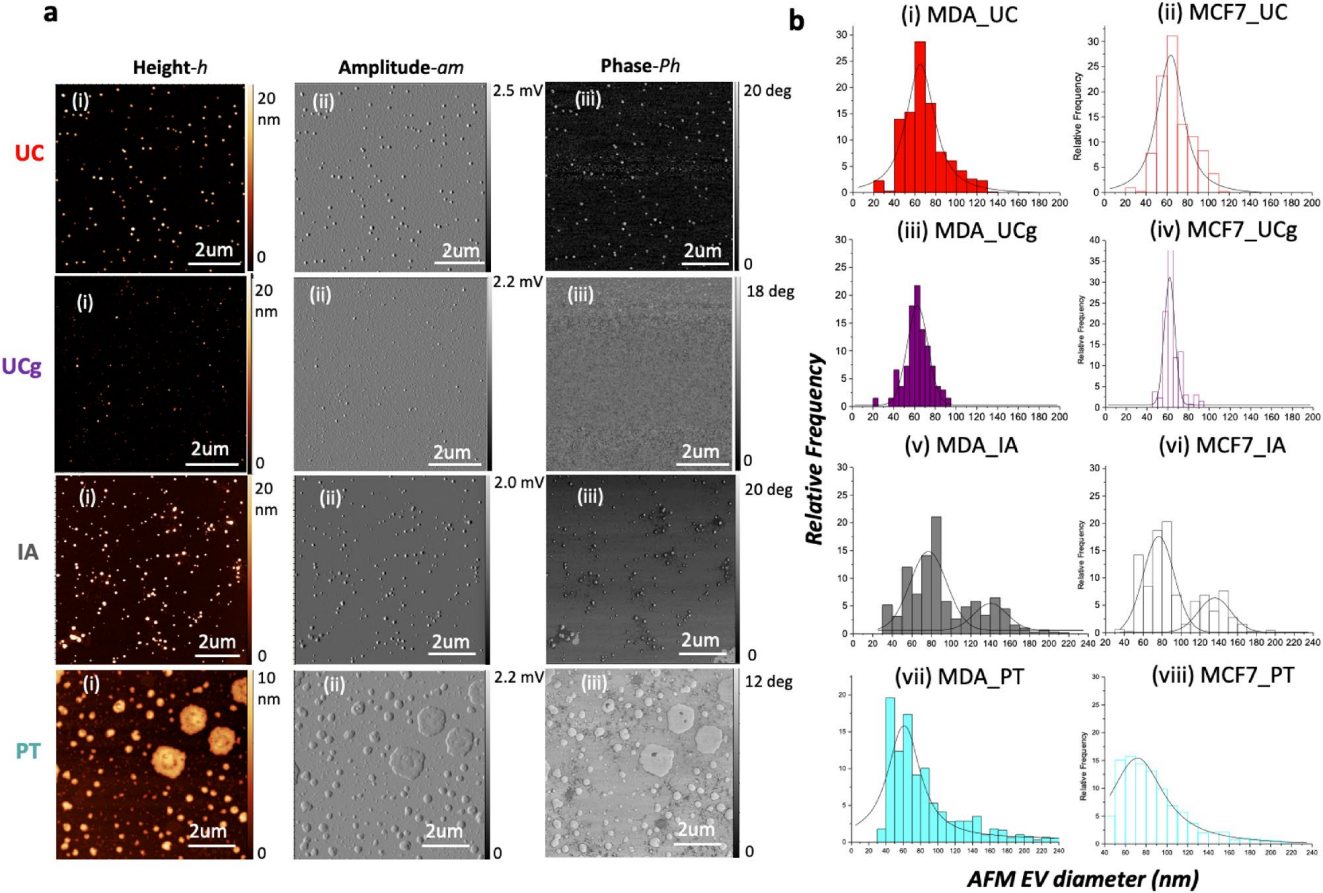
AFM based structural characterization of single sEVs obtained from different isolation methods. (**a**) AFM topographic height*-h* (i), amplitude*-am* (ii) and phase*-Ph* (iii) images for sEVs using UC, UCg, IA and PT isolations are shown from top to bottom respectively. AFM images exhibit least, and highest particle counts per micron square for UCg and PT isolation methods respectively. (**b**) Particle size distribution histograms with Gaussian fits obtained from AFM topography images. While minimal variance in particle size distributions was observed for UCg and largest variations for PT isolates, IA shows two distinct particle size populations, consistent among both cell types (MCF7 and MDA-MB-231), that were found to be significant based on two-way ANOVA (*p < 0.05).

First, we visualized sEVs from four different isolation methods (UC, UCg, IA, and PT as outlined in Fig. 1) from well-established breast cancer cell lines^29^ (described, as sEV_MCF7_ and sEV_MDA-MB-231_ respectively) at the single-particle level using AFM imaging under ambient conditions. The sEVs adsorbed on mica exhibit flat, roundly shaped morphologies. Simultaneously obtained AFM height (Fig. 2a*-h*), amplitude (Fig. 2a*-am*), and phase images (Fig. 2a-*Ph*) emphasize differences in surface topography, standard deviation of topography, and contrast associated with biophysical properties such as elasticity respectively, among the different EV isolates are shown in corresponding panels (Fig. 2; left to right: i–iii). As illustrated in Fig. 2, the overall architecture observed in UC, UCg, and IA isolates reveal frequently occurring circular sub-100 nm particle populations. However, in striking morphological contrast, PT isolates under identical imaging conditions showed relatively large and more heterogeneous particles (> 100 nm) exhibiting irregular morphologies, as shown in representative images (Fig. 2a). At this magnification, no significant substructures were detected on the EV surfaces (AFM*-h, -am*), however, distinct phase differences (AFM-*Ph*) observed for PT isolates indicate high likelihood of presence of polymeric residues on the nanoscale surface features of sEVs (Fig. 2) compared to sEVs from other isolation methods (i.e., UC, UCg, IA).

Further, we determined AFM topography-based particle size (Fig. 2b) and counts, i.e., the number of particles imaged per square micron, in various sEV isolates (summarized in Table 1). Particles displaying a minimum threshold topographic height (> 1 nm), circularity with aspect ratio < 0.5, and a characteristic phase shift under amplitude-modulated AFM, were measured to minimize the influence of any soluble proteins or lipoprotein aggregates. Higher particle counts were noted for MDA-MB-231 compared to MCF7 cell-derived sEVs across three independent isolation techniques (UCg, UC, and IA). In addition to overall higher counts as described using different techniques^5^, and consistent with excess sEVs known to be shed by metastatic cancer cells, AFM particle analysis further resolved the distinct modalities of the size distributions among the four isolation methods. The UC and UCg methods exhibited unimodal distributions but minimal particle size variance, suggesting more homogeneously sized particle populations. The PT method exhibited unimodal but broader, heterogeneously size distributions. In contrast, the IA method exhibited more complex bimodal distributions showing two distinctly sized particle populations. These findings were consistent among sEVs derived from both cell types (i.e., sEV_MCF7_ and sEV_MDA-MB-231_) analyzed in three independent experiments. For each experimental run, cell culture supernatants from ~ 10^6^ cells were split and processed in parallel for the four sEV isolation methods probed (Fig. 1).

#### Particulate to the non-particulate ratio in sEV isolates

**Figure 3:**
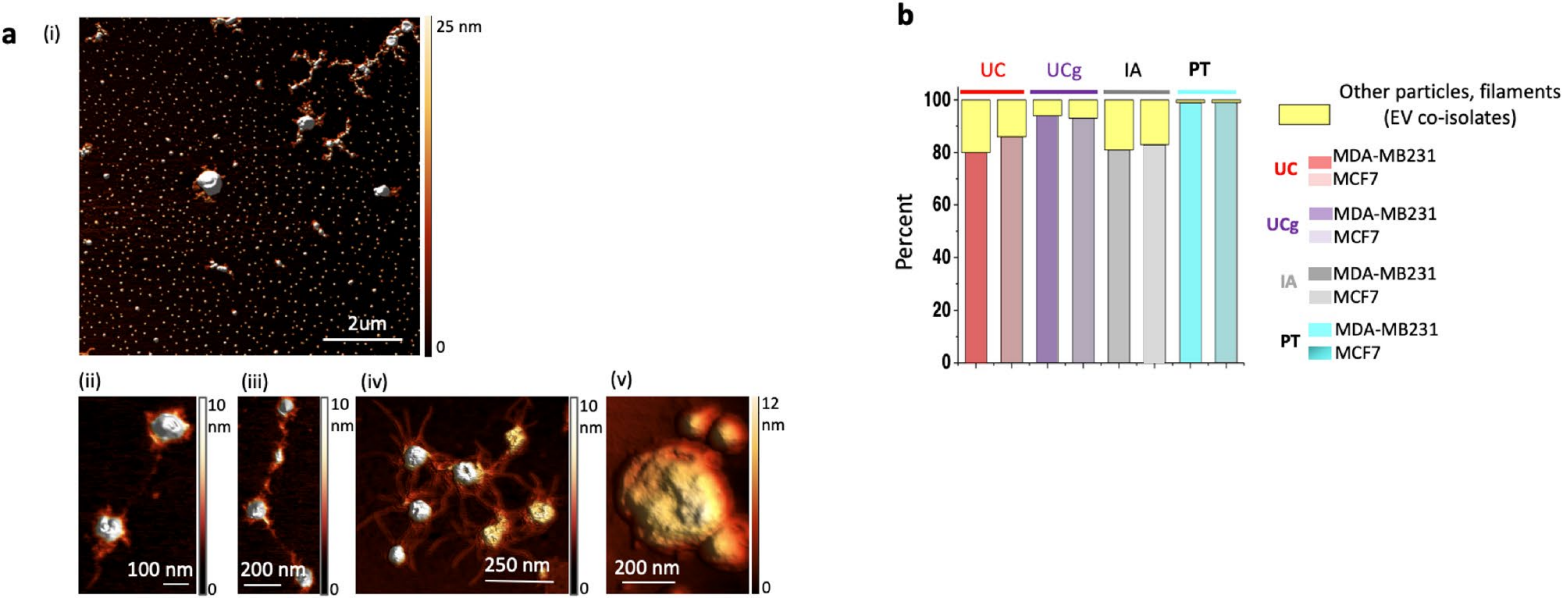
Higher resolution structural analysis of sEV isolates obtained from different isolation methods show the presence of other particles, non-particles and filaments (together called EV co-isolates), at varying abundances. (**a**) Higher resolution AFM images of sEV isolates reveals majority of round circular sEV particles but also structurally diverse sEV co-isolates illustrated in (i-v). Examples of nanoscale filamentous extensions and larger (> 200 nm) particles are shown (**b**) Relative abundances of sEV co-isolates from breast cancer cells varied among different isolation methods.

Second, we employ enhanced resolution AFM imaging as a means to structurally fingerprint and differentiate the sEV isolate components among the various isolates. Most nanoparticle sizing techniques extrapolate particle sizes assuming the spherical shape of the particles within sEV isolates, in addition to an inherent lack of sensitivity to detect additional non-vesicular structures that frequently exist on or associated with sEVs^30^. Here, from a structural standpoint, we used the high-resolution, label-free imaging analysis to biophysically characterize sEV isolates regardless of form and size. Not surprisingly, a systematic survey of sub-nanometer-resolution AFM images of sEVs obtained from different isolation methods revealed the co-existence of nano-sized particles, alongside additional varying filamentous structures albeit at varying abundance based on the isolation methods employed. A few such examples are illustrated in Fig. 3a. Relative ratios of particles (ranging from 10–1000 nm in size) and filamentous components detected in different sEV isolates are quantified in Fig. 3b. The presence of relatively small but reproducible (experimental triplicates) populations of filaments and large (< 500 nm in size) vesicles in UC, and IA sEV isolates for both cell types were noted, which were minimal in case of UCg isolates. In contrast, PT isolates frequently showed large aggregates for both cell types that obscured further structural distinction of constituents within the complexes. The origins or functional relevance of identified co-isolates need to be further investigated. Nevertheless, high-resolution structural determination and quantification of non-vesicle components enabled orthogonal pre-analytical purity assessment criteria for sEV isolates, irrespective of the heterogeneous size, structure, or need for molecular labels.

#### Surface nano-roughness of sEV isolates

**Figure 4:**
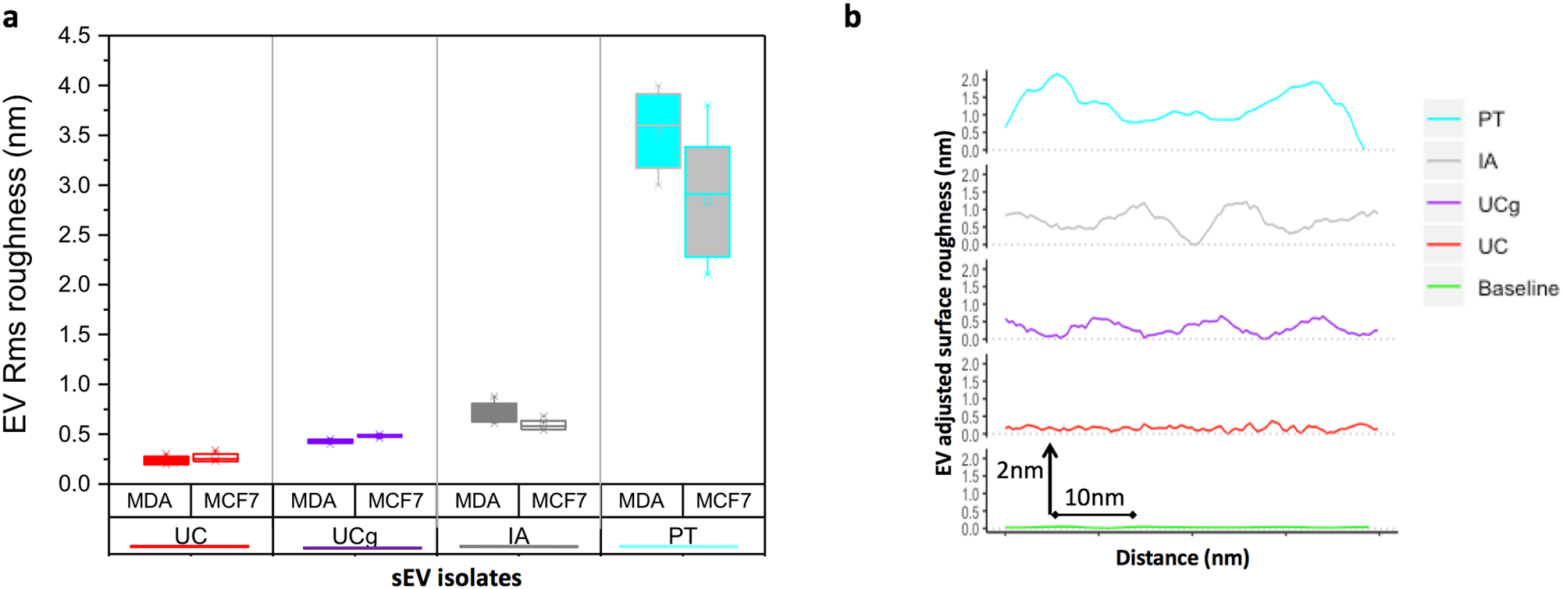
Isolation method influences nanoscale surface topography of sEV isolates. The impact of EV isolation methods on the surface topography of single sEVs was quantified as RMS roughness obtained from AFM topographic images. (**a**) Assessment of RMS roughness of different isolates reveal greatest surface undulations in PT derived sEVs for both MCF7 and MDA-MB-231 cells. (**b**) Representative roughness cross-sectional profiles illustrate variations in surface roughness of different sEVs. Over thirty single sEVs were analyzed for each cell type and isolation method under identical imaging conditions, resolution of topographic images obtained, using identical imaging probes.

Another striking yet underutilized biophysical feature of sEVs is their surface topography. Owing to the relatively large surface to volume ratios with key bio-interfacial roles, surface interactions of sEVs affect different biological and analytical processes^31^. Expanding on our previous findings^32^, we evaluated the topographic root-mean-square (RMS) nano-roughness of individual sEVs, representing the standard deviation of the height profile belonging to the surface asperities (Fig. 4). Our data demonstrate substantial isolation-method dependent variations in RMS nano-roughness of sEVs in breast cancer cells. The RMS nano-roughness of sEVs decreased in the order from PT > IA > UCg > UC isolates for both sEV_MCF7_ and sEV_MDA-MB-231_ (Fig. 4; Table 1). Among these, the highest average roughness values of 3.1 nm and 2.9 nm were observed for PT isolated sEV_MCF7_ and sEV_MDA-MB-231_ respectively. Taken together, the sub-nm resolution mapping of the three-dimensional surface topology of individual sEVs offered a novel metrics to quantify the isolation-method influenced surface complexity of single sEVs with potential implications for biological and analytical behaviors of sEVs that primarily elude typical approaches confined to sEV particle size distribution analysis.

### Comparative optical, light scattering, and microfluidics-based resistive pulse sensing particle size measurements of sEV isolates

**Figure 5:**
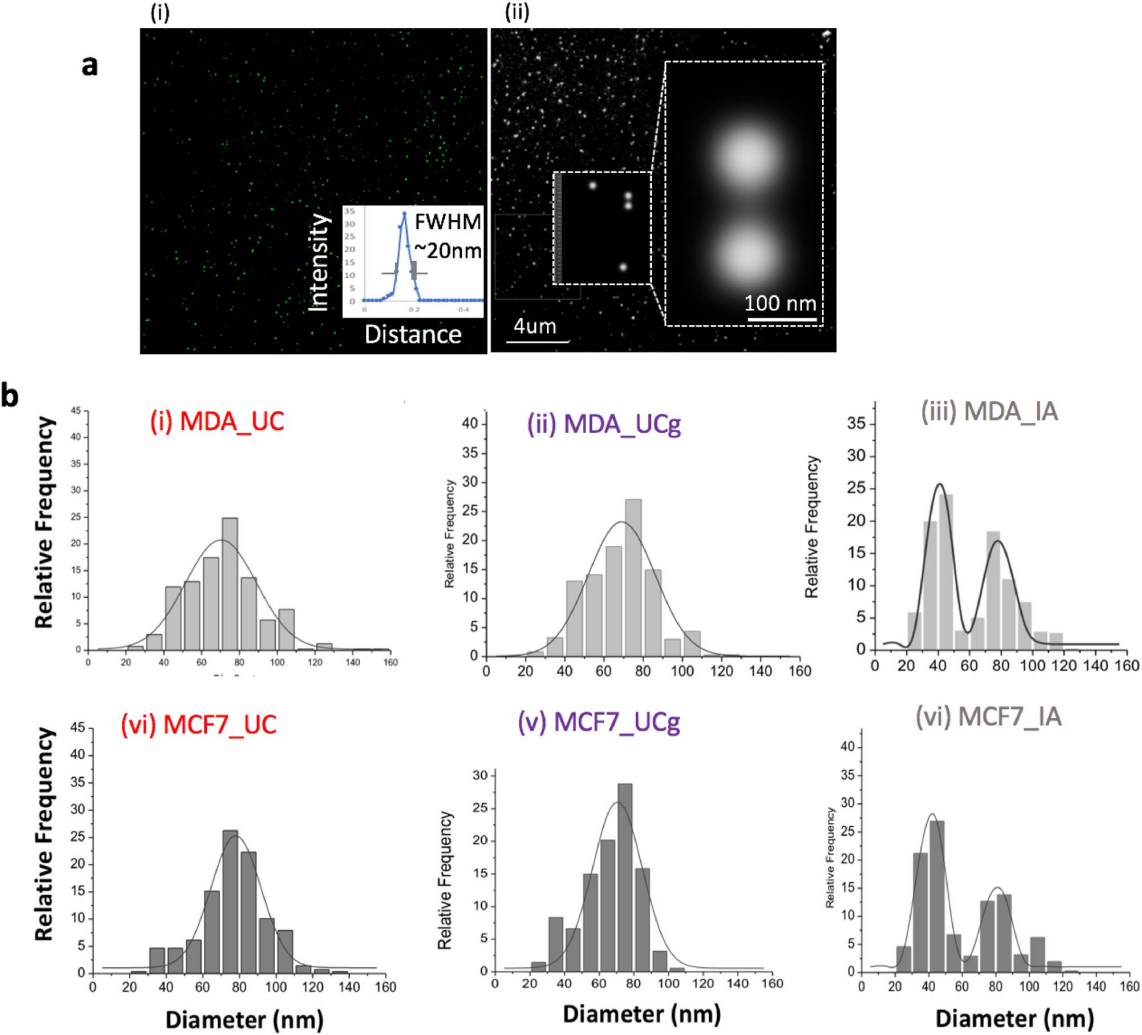
The dSTORM* based particle size characterization of single sEVs obtained from different isolation methods. (**a**) Representative super-resolution dSTORM reconstructed image showing individual lipid labeled sEVs. (i) The full-width half-maximum (FWHM) of the fluorescence intensity distribution ~ 20 nm obtained for dSTORM imaging, ~ 10 times better resolution compared to conventional fluorescence imaging (ii) Zoom-in and inset view showing two well-resolved sEVs, ~ 100 nm in size. (**b**) Histograms showing the size distributions of particles with Gaussian fits obtained for various isolation methods and breast cancer cell types. *direct stochastic optical reconstruction microscopy.

**Figure 6:**
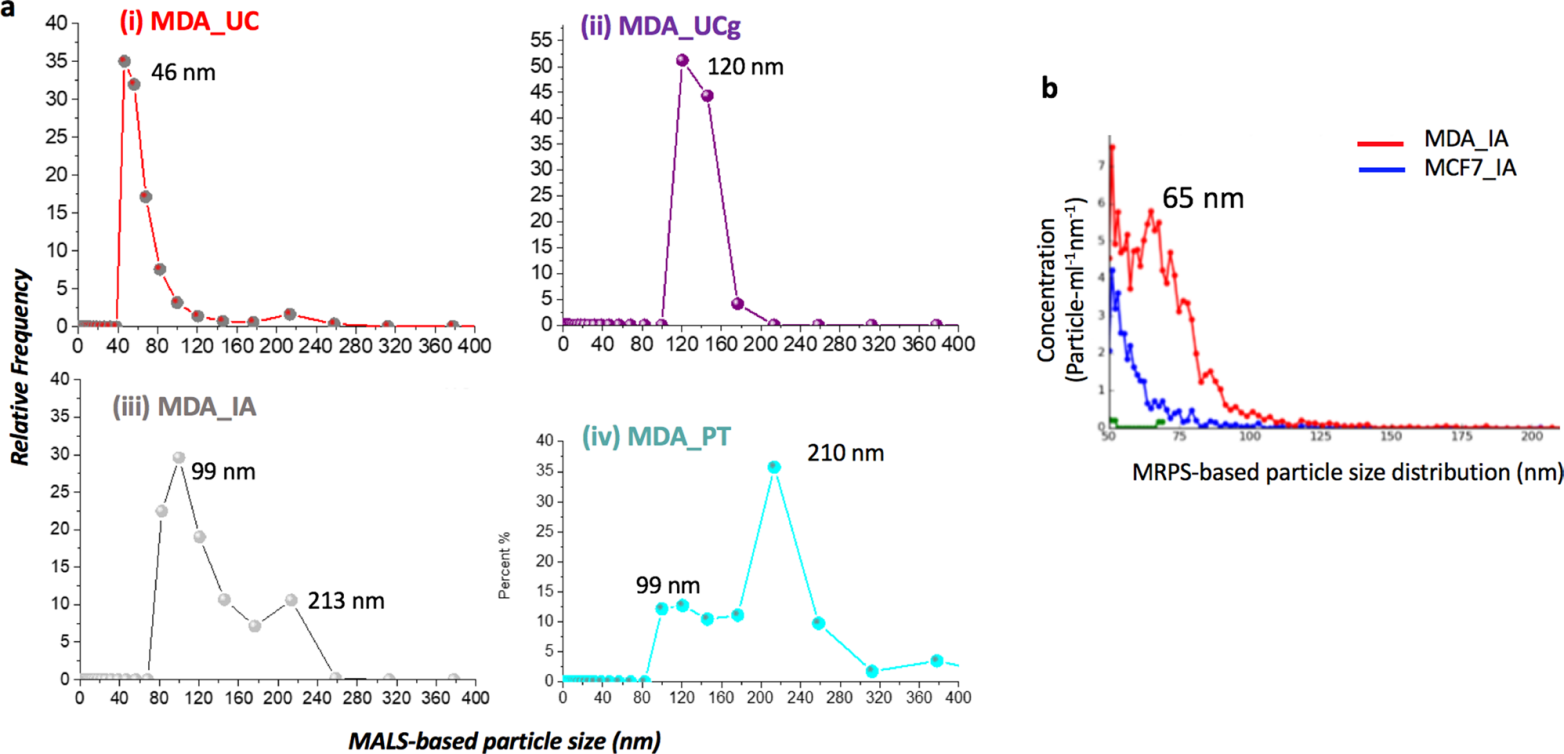
Comparative particle size characterization of breast cancer cell sEVs isolates from different isolation methods via (**a**) MALS based root-mean square radius (Rg) analysis showing averaged results from duplicate runs of each sample. (**b**) Microfluidic resistive pulse sensing (MRPS) showed clear differences between particle concentrations and size distributions among MCF7 and MDA-MB-231 sEVs isolated using IA method within the 50-300 nm size range.

The localization super-resolution microscopy techniques now enable the resolution of light microscopy down to a few tens of nanometers- a scale closer to the typical lateral resolution of AFM when imaging sEVs. This provides an opportunity to contrast the impact of isolation methods on sEVs between scanning probe and optical detection techniques. Therefore, we exploited the resolution capabilities of direct stochastic optical reconstruction microscopy (dSTORM) imaging^33^, to further assess particle size distributions from all four sEV isolation methods and two cell lines studied. A representative reconstructed dSTORM image showing individual lipid labeled sEV nanoparticles is shown in Fig. 5. The measured particle sizes (mean ± Std.dev) for sEVs are reported in Table 1. Overall, the UC and UCg isolation methods displayed unimodal Gaussian distributions for both sEV_MCF7_ and sEV_MDA-MB-231_ (Fig. 5b). In contrast, the IA method showed bimodal size distributions for both the cell types measured i.e., sEV_MCF7_ and sEV_MDA-MB-231_, respectively indicating the presence of two major sub-populations distinctly differing in particle sizes. Thus, the distinct EV subpopulations in case of IA method were independently noted from both AFM and dSTORM particle size distribution analyses. However, the bimodal particle size distributions were undetectable when probed using intensity-weighted light scattering MALS (Fig. 6a) analysis run in parallel on similar samples, despite considerable shifts observed in particle distributions towards larger particle sizes (> 200 nm). We further assessed nanoparticle-sensing technologies based on resistance measurements, in an attempt to better resolve size distributions via particle-by-particle readouts, particularly in case of IA method derived sEV isolates, while requiring a lower sample concentration (~ 10^7^ particles/mL) and smaller sample volume (~ 40 μL). To assess diverse sEV populations independent of light scatter measurements, we probed IA sEV isolates (sEV_MCF7_; and sEV_MDA-MB-231_) using MRPS^34^ (Fig. 6b). The MRPS based particle concentrations, which correspond to the density of particles per mL of solution per nm of particle diameter (particles/mL nm) are displayed in Fig. 6b. The IA method exhibited four times higher particle counts in MDA-MB-231 compared to MCF7 sEV isolates. Very few particles were detected in UC isolates (shown in green curve in Fig. 6b). The higher number of particles noted in IA isolated sEV_MCF7_ compared to sEV_MDA-MB-231_ concurred with both AFM and dSTORM analysis. However, only a single peak particle size ~ 65 nm was observed on repeat MRPS analysis. Together, the multitude of particle size distribution findings on parallel EV isolates demonstrate that single particle AFM and dSTORM approaches enable better resolution and quantification of sEVs than MALS or MRPS alone, and to determine heterogeneity that presumably represent sEV sub-populations or other co-isolates.

## Discussion

Isolation methods impact the biophysical characteristics of sEV isolates at the nanoscale level but elude detection when sEV isolates are assessed based on the particle counts and size distributions alone. Previous studies from our group and others demonstrated the applicability of correlative AFM, EM, and single-molecule force spectroscopy techniques as valuable tools for single sEV biophysical characterization^28,35–38^. Additionally, AFM based nanoscale imaging has been successfully applied to improve quantitative understanding of the impact of isolation methods on surface topographies of sEVs^32,39^ with significant implications for downstream proteomic or transcriptomic assays^8,40–42^. When employing AFM based single particle analysis, the influence of key factors such as AFM imaging mode, magnitudes of imaging forces applied, and the choice of probes and substrates need to be carefully examined^32,43–47^. In this paper, we have conducted a systematic multi-dimensional comparative analysis (between cell types and isolation methods) of in vitro cancer cell-derived sEVs isolates using breast cancer cell models. Direct, high-resolution sEV surface features, quantification of sEV co-isolates, and particle size distributions using multiple sizing techniques were employed to improve our current understanding of nanoscale variations in single sEVs. In contrast to the biological fingerprint of sEVs, we focused on label-free biophysical fingerprinting of sEVs to evaluate the impact of isolation methods at the single-particle level.

Our study introduced nanoscale biophysical characteristics of label-free sEV isolates that differ among four main isolation methods. First, we show variable particle counts and sizes detected among different sEV isolation methods and cell types. The AFM based particle counts reduced from IA > UC > UCg for both sEV_MCF7_ and sEV_MDA-MB-231_ (Table 1). The highest particle counts were detected among PT isolates. However, when probed individually (Fig. 2) the large (> 150–200 nm in diameter) irregular structures and presence of polymeric residues observed via AFM imaging in these sEV isolates reflect the limitations and uncertainties associated with the quality of sEVs in the absence of direct structural mapping. More interestingly, the sEV isolates showed unimodal particle size distribution probabilities, except in the case of IA where the bimodal distributions of sEV_MCF7_ and sEV_MDA-MB-231_ were determined. The AFM-based bimodal distributions of IA derived sEV_MCF7_ and sEV_MDA-MB-231_ were found to be strikingly coherent with findings from the dSTORM particle size determinations, showing reproducible bimodal distributions among the sEV isolates. While MRPS noted about fourfold higher particle counts for sEV_MDA-MB-231_ for IA isolates, the two size populations were not distinguished either via MALS or MRPS based analysis (Fig. 6). While AFM mapped the surfaces of sEV isolates, dSTORM provided complementary information via lipid labeling to reveal particle sizes. The structural mapping of single sEVs thus provides a mechanism to de-convolute isolation method dependent sEV yields (quantity) that may be associated but not necessarily synonymous with greater isolation efficiency (quality).

Second, as a label-free approach, AFM based structural fingerprinting allowed differentiating the sEV isolate components (few nanometers–1000 nm) among the various isolation methods studied. We envision that coupling of high-speed AFM-correlative dSTORM imaging of sEV isolates with machine learning algorithms^48^ for multi-parametric data analysis, such as simultaneous sub-nanometer resolution structural mapping of single sEVs while spatially locating the fluorescently labeled molecules, offers exciting new directions in EV metrology.

Further, we illustrate the applicability of RMS roughness to the repertoire of single sEV biophysical characteristics to provide enhanced inter-methods comparisons of the residual impurities or co-isolates. The sub-nm resolution of z-height measurements in AFM enabled quantitative analysis of surface undulations, separate from the overall particle shapes or sizes. Topographic nano-surface roughness of single sEVs revealed significantly higher surface undulations in IA, and UCg isolates compared to UC. The RMS nano-roughness of sEVs decreased in the order from PT > IA > UCg > UC isolates for both sEV_MCF7_ and sEV_MDA-MB-231_ (Fig. 4; Table 1). The greater than average roughness values of 3.1 nm and 2.9 nm observed for PT isolated sEV_MCF7_ and sEV_MDA-MB-231_ respectively compared to other methods, together with the visualization of aggregates in high-resolution images, strongly suggest that PT isolation results in localization of residual polymeric matrix on sEV surface^39^. Nonetheless, our findings demonstrate the usefulness of orthogonal particle shape/size independent sEV RMS roughness that may be particularly suitable for higher stringency assessment of sEVs isolated from various isolation methods. It is important to note that sEV surfaces are associated with numerous complexes interactions with proteins and other molecules within the dynamic environment (e.g., medium pH, ionic strength or biofluids). The quantitative determination of sEV surface characteristics such as RMS roughness may thus be vital for downstream applications of sEVs including as biomarkers, for functional assays or as therapeutics.

## Conclusions

In summary, the rigorous biophysical characterization of sEVs is a crucial step for their use in diagnostic and clinical medicine. Our work highlights the limitations of comparing sEV isolates based primarily on particle counts and size distributions. Further, we show that correlative and spatially mapped particle metrics (topography, RMS roughness, particle ratios) add unique dimensions for more rigorous and reproducible biophysical characterization of sEVs, overcoming the limitations of conventional particle sizing approaches. The introduction of nanoscale structural characteristics of EV isolates represents a label-free, orthogonal framework to resolve differences in the heterogeneity and purity of sEVs from different cell types and isolation techniques. To the best of our knowledge, this is the first report on correlative scanning probe, optical super-resolution imaging in conjunction with commercial single particle counters to assess the nanoscale impact of sEV isolation methods. These results promise enhanced opportunities for quantitatively evaluating the biophysical quality and purity of sEV isolates that are urgently needed for more reliable sEV based research and clinical utility.

## Materials and methods

### Cell culture and isolation of EVs

To examine the biophysical characteristics of secreted exosomes, we first isolated exosomes from cultured cells that represent different metastatic potentials. We chose MCF-7 and MDA-MB-231 cells, two well-established breast cancer cell lines^29,49^. Also see Supplementary Information for characterization of cells and EVs. MCF-7 cells are tumorigenic but non-metastatic and represent the low metastatic potential. MDA-MB-231 cells are highly metastatic, with altered adhesion and motility properties.

### Small EV (sEV) isolation

The MCF-7 and MDA-MB-231 (ATCC) breast cancer cell lines were cultured in Dulbecco’s modified eagles medium (DMEM; Gibco, Thermo Fischer Scientific, Carlsbad CA) supplemented with 10% fetal bovine serum (FBS; Atlanta Biologicals), 100U/mL penicillin, and 100 μg/mL streptomycin, in a 5% CO_2_ humidified atmosphere at 37 °C. Cells were cultured in six 60 mm Petri dishes (Corning, CA) with FBS-originated-exosome-free media (as per protocol by Théry; FBS was ultra-centrifuged at 100,000×*g* for 2 h at 4 °C, then filtered with a 0.22 µm sterile filter). After 48 h incubation, the media containing sEVs were isolated. Total cell count was 2 × 10^7^ and 24 mL of sEV-containing media was obtained. sEV isolation was performed as outlined in Fig. 1. Successful sEV isolation was confirmed by electron microscopy, immune labeling (CD63, CD81 and CD9), and enrichment of sEV associated proteins determined using Mass Spectrometry (Supplemental Information).

#### Ultracentrifugation (UC)

As outlined in Fig. 1, the sEV-containing media was centrifuged at 2,000×*g* for 20 min at 4 °C to remove cells and other debris. Subsequently, the isolated supernatant (supernatant 1) was centrifuged at 10,000×*g* for 30 min at 4 °C to remove large EVs and supernatant 2 was carefully isolated. To isolate sEVs, supernatant 2 was ultra-centrifuged at 100,000×*g* (type 70 Ti rotor in a Beckman Coulter Optima L-100 XP ultracentrifuge, Beckman Coulter, Inc. USA) for 2 h at 4 °C and supernatant 3 was discarded. The pellet containing sEVs was re-suspended in 1 mL PBS, ultra-centrifuged at 100,000×*g* for 1 h at 4 °C, and supernatant 4 was discarded. Purified sEVs were re-suspended in 1 mL of PBS and stored at 4 °C for subsequent imaging and analysis within < 1 week.

#### Density gradient ultracentrifugation (UCg)

sEV isolation was performed using sucrose cushion as described previously^7^. Briefly, 0.5 ml 30% sucrose solution in PBS was carefully layered beneath 2.5 mL supernatant 2 in an ultracentrifuge tube, and was ultra-centrifuged at 100,000×*g* using a type 70 Ti rotor in a Beckman Coulter Optima L-100 XP ultracentrifuge (Beckman Coulter, Inc., USA) for 2 h at 4 °C. The top 2.5 mL of solution was discarded, and the 30% sucrose bottom layer (0.5 mL) was collected, re-suspended in 3 mL PBS, and the mixture was spun at 100,000×*g* for 1 h at 4 °C, and the resulting supernatant was discarded. Purified sEVs were re-suspended in 1 mL of PBS and stored at 4 °C (< 1 week).

#### Immune affinity (IA)

As per previously described techniques^32^, magnetic immune affinity beads (JSR Life Science, Tokyo, Japan) coated with CD63, CD81 and CD9 antibodies were used to isolate sEVs from cell culture supernatants. Briefly, the sEV-containing media (200 µL) were incubated with 100 µL of capture beads for 60 min at room temperature (RT) with gentle shaking. Using magnets, beads were separated from the supernatant and washed three times using 0.5 mL of wash buffer; beads were gently re-suspended in 50 µL of elution buffer, then incubated without mixing for 3 min at RT. Beads were magnetically removed and the supernatant was diluted to 1 mL with PBS and then dialyzed against PBS (using Slide-A-Lyzer Dialysis Cassette, Thermo Fisher Scientific, CA). Isolated sEVs were stored at 4 °C and analyzed within a week.

#### Precipitation (PT)

Commercial reagents for sEV isolation by PEG precipitation—ExoQuick (System Biosciences, CA) were used according to the manufacturer guidelines. Briefly, 200 µL of MCF7 or MDB-231 sEVs-containing media was combined with 1 mL ExoQuick-TC Solution in sterile Eppendorf tubes, mixed by inverting the tube several times, and incubated at 4 °C for 18 h. The solution was centrifuged at 1,500×*g* for 30 min in an Eppendorf MiniSpin Plus (Eppendorf, Germany). The supernatant was discarded, and the pellet was suspended in 1 mL PBS and stored at 4 °C.

### AFM imaging, particle size, and single particle nano-roughness analysis

sEV samples were incubated on freshly cleaved mica substrates (TedPella Inc, CA) for 5 min, washed with de-ionized water to remove any unbound EVs and air-dried overnight. Samples were imaged by Dimension Icon (Bruker Instruments, CA, USA) using the amplitude mode via TESP probes (Bruker Instruments, CA, USA) and images were recorded at 1,024 samples per line at 1 Hz.

In our study, AFM imaging of isolated sEVs was performed at room temperature (22 ± 1 °C) and ambient humidity ranging from 40 to 45% relative humidity for all samples studied. Both temperature and humidity (with impact on factors such as capillary forces, friction, lubrication) play significant role in local-tip sample interactions. While the detailed influencing mechanisms of environment conditions such as temperature and humidity, were beyond the scope of these current investigations, the use of controlled environmental chamber enclosing the sample and the probe should be carefully considered, especially for metrological applications of AFM for EV analysis.

Image processing was done using SPIP (Image Metrology, Denmark) software to quantify particle diameters and counts using grain analysis function. For sEV yield calculation, images at sizes of 1 µm × 1 µm and 8 µm × 8 µm at a resolution of 1,024 samples per line, at 1 Hz, were used. As per previously described for glioblastoma cell line derived EVs, to evaluate surface nano-roughness we employed 1 µm × 1 µm AFM topographic images^35^. The z (x, y) profiles, exported from AFM topographic scans were plane flattened to mitigate the effects of mostly curved nature of the nanoparticles. The apical regions (sub-tending typically 10% of the total area) were fitted with smooth spherical profiles, using least squares regression to obtain the average radius and center location for each nanoparticle (See Supplemental Information). Subsequently, the radial component of the spherical fit was subtracted from the distance between the center of the smooth profile and each (x, y, z) surface location. The resulting radial roughness amplitudes (Fig. 4) were then used to calculate RMS for single sEVs^50^. Casein micelles from bovine milk without and without protease treatment were used to validate the quantitative nano-roughness analysis of single particles using AFM analysis.

### dSTORM image acquisition and data analysis

#### Sample preparation

Isolated sEVs were labeled with 0.0025 mM carbocyanine dye (Vybrant DiO, Molecular Probes, OR) in PBS^51^. Samples were incubated over poly-lysine coated glass coverslips at RT for 20 min and washed twice before imaging. The *d*STORM^52^, acquisition was performed in an oxygen-scavenger buffer solution (ethanolamine, OxyFluor, and DL-Lactase adjusted to pH 7.5–8.5. A total internal reflection fluorescence microscope (Leica GSD SR, IL, US) with a 100 × oil-immersion objective featuring a numerical aperture of 1.46 was used^51^. Lasers 405 nm and 642 nm were used for activation and excitation of DiO, respectively. For a single acquisition, 20,000 images of 32.56 × 32.56 µm were recorded with a cooled, electron multiplying charge-coupled device camera (Leica, IL, US) using 50 ms for exposure time. Calibration experiments were done with known size fluorescent beads (20 and 100 nm). EV-free samples [DiO in PBS] used as controls indicated no detectable (or < 100 times lower) photo-blinking events in the far-red channel, suggesting the absence of non-specific fluorescence from the unbound dye. The images were recorded using Leica software (LAX Life Sciences).

### Micro-fluidic resistive pulse sensing (MRPS)

For MRPS measurements, the supernatants and all solutions were filtered through a 0.2 μm filter (Millipore) prior to use, as per the manufacturer’s recommendation. Measurements were performed using an nCS1 machine (Spectradyne LLC, Torrance, CA) utilizing single use poly-dimethylsiloxane cartridges (TS-400) with pore size ranging from 65–400 nm (as per the manufacturer’s manual). According to the manufacturer’s recommendation, about three micro-liters of filtered sEV supernatants were used for each measurement and at least five-hundred individual events (particles) were assessed for analysis. Each cartridge was calibrated using standard polystyrene beads (100 ± 2 nm in size). Data were analyzed via nCS1 Data Analyzer (Spectradyne LLC, Torrance, CA) and employed similar user-defined transit time, signal-to-noise ratios and characteristics peak setting across all samples analyzed. The particle size and counts were determined using the average of triplicate measurements on two independent samples.

### Dynamic and multi-angle laser light scattering (MALS) for particle size determination

Using Dyna Pro (Wyatt Technologies, Santa Barbara, CA), sixty micro-liters of sEV samples were analyzed in triplicates to obtain *R*g and absolute nanoparticle concentrations. Light scattering measurements were taken continuously at 18 angles between 15° and 151°; the captured data were integrated and analyzed using the DYNAMICS software (Wyatt Technologies).

## Supplementary information

Supplementary information

## Data Availability

The data that support the findings of this study are available from the corresponding author upon reasonable request.

## References

[1] Théry C (2011). Exosomes: secreted vesicles and intercellular communications. F1000 Biol Rep.

[2] Xu R, Rai A, Chen M, Suwakulsiri W, Greening DW, Simpson RJ (2018). Extracellular vesicles in cancer—implications for future improvements in cancer care. Nat. Rev. Clin. Oncol..

[3] Théry C, Witwer KW, Aikawa E, Alcaraz MJ, Anderson JD, Andriantsitohaina R, Antoniou A, Arab T, Archer F, Atkin-Smith GK, Ayre DC, Bach J-M, Bachurski D, Baharvand H, Balaj L, Baldacchino S, Bauer NN, Baxter AA, Bebawy M, Beckham C, Zavec AB, Benmoussa A, Berardi AC, Bergese P, Bielska E, Blenkiron C, Bobis-Wozowicz S, Boilard E, Boireau W, Bongiovanni A, Borràs FE, Bosch S, Boulanger CM, Breakefield X, Breglio AM, Brennan MÁ, Brigstock DR, Brisson A, Broekman ML, Bromberg JF, Bryl-Górecka P, Buch S, Buck AH, Burger D, Busatto S, Buschmann D, Bussolati B, Buzás EI, Byrd JB, Camussi G, Carter DR, Caruso S, Chamley LW, Chang Y-T, Chen C, Chen S, Cheng L, Chin AR, Clayton A, Clerici SP, Cocks A, Cocucci E, Coffey RJ, Cordeiro-da-Silva A, Couch Y, Coumans FA, Coyle B, Crescitelli R, Criado MF, D’Souza-Schorey C, Das S, Chaudhuri AD, de Candia P, Junior EFDS, Wever OD, del Portillo HA, Demaret T, Deville S, Devitt A, Dhondt B, Vizio DD, Dieterich LC, Dolo V, Rubio APD, Dominici M, Dourado MR, Driedonks TA, Duarte FV, Duncan HM, Eichenberger RM, Ekström K, Andaloussi SE, Elie-Caille C, Erdbrügger U, Falcón-Pérez JM, Fatima F, Fish JE, Flores-Bellver M, Försönits A, Frelet-Barrand A, Fricke F, Fuhrmann G, Gabrielsson S, Gámez-Valero A, Gardiner C, Gärtner K, Gaudin R, Gho YS, Giebel B, Gilbert C, Gimona M, Giusti I, Goberdhan DC, Görgens A, Gorski SM, Greening DW, Gross JC, Gualerzi A, Gupta GN, Gustafson D, Handberg A, Haraszti RA, Harrison P, Hegyesi H, Hendrix A, Hill AF, Hochberg FH, Hoffmann KF, Holder B, Holthofer H, Hosseinkhani B, Hu G, Huang Y, Huber V, Hunt S, Ibrahim AG-E, Ikezu T, Inal JM, Isin M, Ivanova A, Jackson HK, Jacobsen S, Jay SM, Jayachandran M, Jenster G, Jiang L, Johnson SM, Jones JC, Jong A, Jovanovic-Talisman T, Jung S, Kalluri R, Kano S, Kaur S, Kawamura Y, Keller ET, Khamari D, Khomyakova E, Khvorova A, Kierulf P, Kim KP, Kislinger T, Klingeborn M, Kornek M, Kosanović MM, Kovács ÁF, Krämer-Albers E-M, Krasemann S, Krause M, Kurochkin IV, Kusuma GD, Kuypers S, Laitinen S, Langevin SM, Languino LR, Lannigan J, Lässer C, Laurent LC, Lavieu G, Lázaro-Ibáñez E, Lay SL, Lee M-S, Lee YXF, Lemos DS, Lenassi M, Leszczynska A, Li IT, Liao K, Libregts SF, Ligeti E, Lim R, Lim SK, Linē A, Linnemannstöns K, Llorente A, Lombard CA, Lorenowicz MJ, Lörincz ÁM, Lötvall J, Lovett J, Lowry MC, Loyer X, Lu Q, Lukomska B, Lunavat TR, Maas SL, Malhi H, Marcilla A, Mariani J, Mariscal J, Martens-Uzunova ES, Martin-Jaular L, Martinez MC, Martins VR, Mathieu M, Mathivanan S, Maugeri M, McGinnis LK, McVey MJ, Meehan KL, Mertens I, Minciacchi VR, Möller A, Jørgensen MM, Morales-Kastresana A, Morhayim J, Mullier F, Muraca M, Musante L, Mussack V, Muth DC, Myburgh KH, Najrana T, Nawaz M, Nazarenko I, Nejsum P, Neri C, Neri T, Nieuwland R, Nimrichter L, Nolan JP, Hoen ENN, Hooten NN, O’Driscoll L, O’Grady T, O’Loghlen A, Ochiya T, Olivier M, Ortiz A, Ortiz LA, Osteikoetxea X, Østergaard O, Ostrowski M, Park J, Pegtel DM, Peinado H, Perut F, Pfaffl MW, Phinney DG, Pieters BC, Pink RC, Pisetsky DS, von Strandmann EP, Polakovicova I, Poon IK, Powell BH, Prada I, Pulliam L, Quesenberry P, Radeghieri A, Raffai RL, Raimondo S, Rak J, Ramirez MI, Raposo G, Rayyan MS, Regev-Rudzki N, Ricklefs FL, Robbins PD, Roberts DD, Rodrigues SC, Rohde E, Rome S, Rouschop KM, Rughetti A, Russell AE, Saá P, Sahoo S, Salas-Huenuleo E, Sánchez C, Saugstad JA, Saul MJ, Schiffelers RM, Schneider R, Schøyen TH, Scott A, Shahaj E, Sharma S, Shatnyeva O, Shekari F, Shelke GV, Shetty AK, Shiba K, Siljander PR-M, Silva AM, Skowronek A, Soares RP, Sódar BW, Soekmadji C, Sotillo J, Stahl PD, Stoorvogel W, Stott SL, Strasser EF, Swift S, Tahara H, Tewari M, Timms K, Tiwari S, Tixeira R, Tkach M, Toh WS, Tomasini R, Torrecilhas AC, Tosar JP, Toxavidis V, Urbanelli L, Vader P, van Balkom BW, van der Grein SG, Deun JV, van Herwijnen MJ, Keuren-Jensen KV, van Niel G, van Royen ME, van Wijnen AJ, Vasconcelos MH, Veit TD, Vella LJ, Velot É, Verweij FJ, Vestad B, Viñas JL, Visnovitz T, Vukman KV, Wahlgren J, Watson DC, Wauben MH, Weaver A, Webber JP, Weber V, Wehman AM, Weiss DJ, Welsh JA, Wendt S, Wheelock AM, Wiener Z, Witte L, Wolfram J, Xagorari A, Xander P, Xu J, Yan X, Yáñez-Mó M, Yin H, Yuana Y, Zappulli V, Zarubova J, Žėkas V, Zhang J, Zhao Z, Zheng L, Zheutlin AR, Zickler AM, Zimmermann P, Zivkovic AM, Zocco D, Zuba-Surma EK (2018). Minimal information for studies of extracellular vesicles 2018 (MISEV2018): a position statement of the international society for extracellular vesicles and update of the MISEV2014 guidelines. J. Extracell. Vesicles.

[4] Jeppesen DK, Fenix AM, Franklin JL, Higginbotham JN, Zhang Q, Zimmerman LJ, Liebler DC, Ping J, Liu Q, Evans R, Fissell WH, Patton JG, Rome LH, Burnette DT, Coffey RJ (2019). Reassessment of Exosome Composition. Cell.

[5] Zhang H, Freitas D, Kim HS, Fabijanic K, Li Z, Chen H, Mark MT, Molina H, Martin AB, Bojmar L, Fang J, Rampersaud S, Hoshino A, Matei I, Kenific CM, Nakajima M, Mutvei AP, Sansone P, Buehring W, Wang H, Jimenez JP, Cohen-Gould L, Paknejad N, Brendel M, Manova-Todorova K, Magalhães A, Ferreira JA, Osório H, Silva AM, Massey A, Cubillos-Ruiz JR, Galletti G, Giannakakou P, Cuervo AM, Blenis J, Schwartz R, Brady MS, Peinado H, Bromberg J, Matsui H, Reis CA, Lyden D (2018). Identification of distinct nanoparticles and subsets of extracellular vesicles by asymmetric flow field-flow fractionation. Nat. Cell Biol..

[6] Choi D, Spinelli C, Montermini L, Rak J (2019). Oncogenic regulation of extracellular vesicle proteome and heterogeneity. Proteomics.

[7] Théry C, Amigorena S, Raposo G, Clayton A (2006). Isolation and characterization of exosomes from cell culture supernatants and biological fluids. Curr. Protoc. Cell Biol..

[8] Tauro BJ, Greening DW, Mathias RA, Ji H, Mathivanan S, Scott AM, Simpson RJ (2012). Comparison of ultracentrifugation, density gradient separation, and immunoaffinity capture methods for isolating human colon cancer cell line LIM1863-derived exosomes. Methods.

[9] Böing AN, van der Pol E, Grootemaat AE, Coumans FAW, Sturk A, Nieuwland R (2014). Single-step isolation of extracellular vesicles by size-exclusion chromatography. J. Extracell. Vesicles.

[10] Bobrie A, Colombo M, Krumeich S, Raposo G, Théry C (2012). Diverse subpopulations of vesicles secreted by different intracellular mechanisms are present in exosome preparations obtained by differential ultracentrifugation. J. Extracell. Vesicles.

[11] Maas SLN, de Vrij J, van der Vlist EJ, Geragousian B, van Bloois L, Mastrobattista E, Schiffelers RM, Wauben MHM, Broekman MLD, Nolte Hoen ENM (2015). Possibilities and limitations of current technologies for quantification of biological extracellular vesicles and synthetic mimics. J. Control Release.

[12] Rupert DLM, Claudio V, Lässer C, Bally M (2017). Methods for the physical characterization and quantification of extracellular vesicles in biological samples. Biochim. Biophys. Acta (BBA).

[13] Van Deun J, Mestdagh P, Agostinis P, Akay Ö, Anand S, Anckaert J, Martinez ZA, Baetens T, Beghein E, Bertier L, Berx G, Boere J, Boukouris S, Bremer M, Buschmann D, Byrd JB, Casert C, Cheng L, Cmoch A, Daveloose D, De Smedt E, Demirsoy S, Depoorter V, Dhondt B, Driedonks TAP, Dudek A, Elsharawy A, Floris I, Foers AD, Gärtner K, Garg AD, Geeurickx E, Gettemans J, Ghazavi F, Giebel B, Kormelink TG, Hancock G, Helsmoortel H, Hill AF, Hyenne V, Kalra H, Kim D, Kowal J, Kraemer S, Leidinger P, Leonelli C, Liang Y, Lippens L, Liu S, Lo Cicero A, Martin S, Mathivanan S, Mathiyalagan P, Matusek T, Milani G, Monguió-Tortajada M, Mus LM, Muth DC, Németh A, Nolte Hoen ENM, O’Driscoll L, Palmulli R, Pfaffl MW, Primdal-Bengtson B, Romano E, Rousseau Q, Sahoo S, Sampaio N, Samuel M, Scicluna B, Soen B, Steels A, Swinnen JV, Takatalo M, Thaminy S, Théry C, Tulkens J, Van Audenhove I, van der Grein S, Van Goethem A, van Herwijnen MJ, Van Niel G, Van Roy N, Van Vliet AR, Vandamme N, Vanhauwaert S, Vergauwen G, Verweij F, Wallaert A, Wauben M, Witwer KW, Zonneveld MI, De Wever O, Vandesompele J, Hendrix A (2017). EV-TRACK: transparent reporting and centralizing knowledge in extracellular vesicle research. Nat. Methods.

[14] van der Pol E, Coumans FAW, Grootemaat AE, Gardiner C, Sargent IL, Harrison P, Sturk A, Leeuwen TG (2014). Particle size distribution of exosomes and microvesicles determined by transmission electron microscopy, flow cytometry, nanoparticle tracking analysis, and resistive pulse sensing. J. Throm. Haemost..

[15] Khatun Z, Bhat A, Sharma S, Sharma A (2016). Elucidating diversity of exosomes: biophysical and molecular characterization methods. Nanomedicine.

[16] van der Pol E, Böing AN, Gool EL, Nieuwland R (2016). Recent developments in the nomenclature, presence, isolation, detection and clinical impact of extracellular vesicles. J. Thromb. Haemost..

[17] Pol EVD, Hoekstra AG, Sturk A, Otto C, Leeuwen TGV, Nieuwland R (2010). Optical and Non-Optical Methods for Detection and Characterization of Microparticles and Exosomes. J. Thromb. Haemost..

[18] Morales-Kastresana A, Musich TA, Welsh JA, Telford W, Demberg T, Wood JCS, Bigos M, Ross CD, Kachynski A, Dean A, Felton EJ, Dyke JV, Tigges J, Toxavidis V, Parks DR, Overton WR, Kesarwala AH, Freeman GJ, Rosner A, Perfetto SP, Pasquet L, Terabe M, McKinnon K, Kapoor V, Trepel JB, Puri A, Kobayashi H, Yung B, Chen X, Guion P, Choyke P, Knox SJ, Ghiran I, Robert-Guroff M, Berzofsky JA, Jones JC (2019). High-fidelity detection and sorting of nanoscale vesicles in viral disease and cancer. J. Extracell. Vesicles.

[19] Weatherall E, Willmott GR (2015). Applications of tunable resistive pulse sensing. Analyst.

[20] Gardiner, C.; Ferreira, Y. J.; Dragovic, R. A.; Redman, C. W. G.; Sargent, I. L. Extracellular Vesicle Sizing and Enumeration by Nanoparticle Tracking Analysis. *J Extracell Vesicles* **2013**, *2*. 10.3402/jev.v2i0.19671. 10.3402/jev.v2i0.19671 PMC3760643 24009893

[21] Arraud N, Linares R, Tan S, Gounou C, Pasquet J-M, Mornet S, Brisson AR (2014). Extracellular vesicles from blood plasma: determination of their morphology, size, phenotype and concentration. J. Thromb. Haemost..

[22] Chandler WL, Yeung W, Tait JF (2011). A new microparticle size calibration standard for use in measuring smaller microparticles using a new flow cytometer. J. Thromb. Haemost..

[23] Kormelink TG, Arkesteijn GJA, Nauwelaers FA, van den Engh G, Hoen ENMN, Wauben MHM (2016). Prerequisites for the analysis and sorting of extracellular vesicle subpopulations by high-resolution flow cytometry. Cytometry A.

[24] Pasalic L, Williams R, Siupa A, Campbell H, Henderson MJ, Chen VMY (2016). Enumeration of extracellular vesicles by a new improved flow cytometric method is comparable to fluorescence mode nanoparticle tracking analysis. Nanomedicine.

[25] Pan BT, Teng K, Wu C, Adam M, Johnstone RM (1985). Electron microscopic evidence for externalization of the transferrin receptor in vesicular form in sheep reticulocytes. J. Cell Biol..

[26] Chiang C, Chen C (2019). Toward characterizing extracellular vesicles at a single-particle level. J. Biomed. Sci..

[27] Binnig G, Quate CF, Gerber Ch (1986). Atomic force microscope. Phys. Rev. Lett..

[28] Sharma S, Rasool HI, Palanisamy V, Mathisen C, Schmidt M, Wong DT, Gimzewski JK (2010). Structural-Mechanical Characterization of Nanoparticle Exosomes in Human Saliva, Using Correlative AFM, FESEM, and Force Spectroscopy. ACS Nano.

[29] Neve RM, Chin K, Fridlyand J, Yeh J, Baehner FL, Fevr T, Clark L, Bayani N, Coppe J-P, Tong F, Speed T, Spellman PT, DeVries S, Lapuk A, Wang NJ, Kuo W-L, Stilwell JL, Pinkel D, Albertson DG, Waldman FM, McCormick F, Dickson RB, Johnson MD, Lippman M, Ethier S, Gazdar A, Gray JW (2006). A collection of breast cancer cell lines for the study of functionally distinct cancer subtypes. Cancer Cell.

[30] Tkach M, Kowal J, Théry C (2018). Why the need and how to approach the functional diversity of extracellular vesicles. Philos. Trans. R. Soc. Lond. B. Biol. Sci..

[31] Buzás EI, Tóth EÁ, Sódar BW, Szabó-Taylor KÉ (2018). Molecular interactions at the surface of extracellular vesicles. Semin. Immunopathol..

[32] Woo J, Sharma S, Gimzewski J (2016). The role of isolation methods on a nanoscale surface structure and its effect on the size of exosomes. J. Circ. Biomark..

[33] Hell SW (2007). Far-field optical nanoscopy. Science.

[34] Fraikin J-L, Teesalu T, McKenney CM, Ruoslahti E, Cleland AN (2011). A high-throughput label-free nanoparticle analyser. Nat. Nanotechnol..

[35] Sharma S, Das K, Woo J, Gimzewski JK (2014). Nanofilaments on glioblastoma exosomes revealed by peak force microscopy. J. R. Soc. Interface.

[36] Yuana Y, Oosterkamp TH, Bahatyrova S, Ashcroft B, Garcia Rodriguez P, Bertina RM, Osanto S (2010). Atomic force microscopy: a novel approach to the detection of nanosized blood microparticles. J. Thromb. Haemost..

[37] Ashcroft BA, de Sonneville J, Yuana Y, Osanto S, Bertina R, Kuil ME, Oosterkamp TH (2012). Determination of the size distribution of blood microparticles directly in plasma using atomic force microscopy and microfluidics. Biomed. Microdev..

[38] Siedlecki CA, Wen Wang I, Higashi JM, Kottke-Marchant K, Marchant RE (1999). Platelet-Derived Microparticles on Synthetic Surfaces Observed by Atomic Force Microscopy and Fluorescence Microscopy. Biomaterials.

[39] Paolini L, Zendrini A, Noto GD, Busatto S, Lottini E, Radeghieri A, Dossi A, Caneschi A, Ricotta D, Bergese P (2016). Residual matrix from different separation techniques impacts exosome biological activity. Sci. Rep..

[40] Van Deun J, Mestdagh P, Sormunen R, Cocquyt V, Vermaelen K, Vandesompele J, Bracke M, De Wever O, Hendrix A (2014). The impact of disparate isolation methods for extracellular vesicles on downstream RNA profiling. J. Extracell. Vesicles.

[41] Varga, Z.; Yuana, Y.; Grootemaat, A. E.; van der Pol, E.; Gollwitzer, C.; Krumrey, M.; Nieuwland, R. Towards Traceable Size Determination of Extracellular Vesicles. *J Extracell Vesicles* **2014**, *3*. 10.3402/jev.v3.23298. 10.3402/jev.v3.23298 PMC3916677 24511372

[42] Lozano-Ramos, I.; Bancu, I.; Oliveira-Tercero, A.; Armengol, M. P.; Menezes-Neto, A.; Del Portillo, H. A.; Lauzurica-Valdemoros, R.; Borràs, F. E. Size-Exclusion Chromatography-Based Enrichment of Extracellular Vesicles from Urine Samples. *J Extracell Vesicles* **2015**, *4*. 10.3402/jev.v4.27369. 10.3402/jev.v4.27369 PMC4449362 26025625

[43] Thomson NH (2005). Imaging the substructure of antibodies with tapping-mode afm in air: the importance of a water layer on mica. J. Microsc..

[44] Dokukin ME, Guz NV, Gaikwad RM, Woodworth CD, Sokolov I (2011). Cell surface as a fractal: normal and cancerous cervical cells demonstrate different fractal behavior of surface adhesion maps at the nanoscale. Phys. Rev. Lett..

[45] Sharma S, Gillespie BM, Palanisamy V, Gimzewski JK (2011). Quantitative Nanostructural and Single-Molecule Force Spectroscopy Biomolecular Analysis of Human-Saliva-Derived Exosomes. Langmuir.

[46] Vorselen D, MacKintosh FC, Roos WH, Wuite GJL (2017). Competition between bending and internal pressure governs the mechanics of fluid nanovesicles. ACS Nano.

[47] Calò A, Reguera D, Oncins G, Persuy M-A, Sanz G, Lobasso S, Corcelli A, Pajot-Augy E, Gomila G (2014). Force measurements on natural membrane nanovesicles reveal a composition-independent high young’s modulus. Nanoscale.

[48] Ito K, Ogawa Y, Yokota K, Matsumura S, Minamisawa T, Suga K, Shiba K, Kimura Y, Hirano-Iwata A, Takamura Y, Ogino T (2018). Host cell prediction of exosomes using morphological features on solid surfaces analyzed by machine learning. J. Phys. Chem. B.

[49] Nagaraja GM, Othman M, Fox BP, Alsaber R, Pellegrino CM, Zeng Y, Khanna R, Tamburini P, Swaroop A, Kandpal RP (2006). Gene expression signatures and biomarkers of noninvasive and invasive breast cancer cells: comprehensive profiles by representational difference analysis microarrays and proteomics. Oncogene.

[50] Hsu C-P, Ramakrishna SN, Zanini M, Spencer ND, Isa L (2018). Roughness-dependent tribology effects on discontinuous shear thickening. PNAS.

[51] Heilemann M, van de Linde S, Schüttpelz M, Kasper R, Seefeldt B, Mukherjee A, Tinnefeld P, Sauer M (2008). Subdiffraction-resolution fluorescence imaging with conventional fluorescent probes. Angew. Chem. Int. Ed..

[52] van de Linde S, Löschberger A, Klein T, Heidbreder M, Wolter S, Heilemann M, Sauer M (2011). Direct stochastic optical reconstruction microscopy with standard fluorescent probes. Nat. Protoc..

