# Supplementary material for: Impact of isolation methods on the biophysical heterogeneity of single extracellular vesicles
Source: Sci Rep. 2020 Aug 7;10:13327. doi: 10.1038/s41598-020-70245-1 (PMC7414114; doi:10.1038/s41598-020-70245-1)
Supplement: Supplementary file 1 — Supplementary information [file 41598_2020_70245_MOESM1_ESM.pdf]

Supplemental Information

Impact of isolation methods on the biophysical heterogeneity of single extracellular vesicles

Shivani Sharma*[1,2,3], Michael LeClaire[4], James Wohlschlegel[5], James Gimzewski[2,3,4]

[1]Department of Pathology & Laboratory Medicine, David Geffen School of Medicine at UCLA; [2]California NanoSystems Institute, [3]Jonsson Comprehensive Cancer Center, [4]Department of Chemistry & Biochemistry, [5]Department of Biological Chemistry, David Geffen School of Medicine, University of California Los Angeles, CA 90095

**Corresponding author*

Email: sharmas@ucla.edu

S1: Structural and biomolecular characterization of small EV isolates:

The following key measures were undertaken to minimize hitchhiker contaminations, from interfering with the analysis of EV isolation and characterizations in our study.

1. A prerequisite for any EV-related study is the use of contamination-free cells. Mycoplasma or other microbes can also release vesicles interfering with the purity and characterization of EVs. We employed rigorous testing of any contamination of the cells, cell culture media, other reagents, or chemicals. Cell cultures (ATCC) were regularly tested and found negative for mycoplasma contamination using the culture medium (Mycoplasma broth, Millipore Sigma) and microscopic analysis.
2. In case of cell culture derived sEVs, of major importance are medium components, which are highly enriched in proteins and contain EVs, such as fetal bovine serum (FBS). Taking into account that sEVs present in FBS that reportedly co-isolate with cultured cells, we used EV depleted media. The EV-free media was also evaluated microscopically to validate the depletion of EV sized particles. Further, since a full depletion of EVs may not be possible, as per the recommendations of the ISEV guidelines (Thery et al. JEV 2018), we used fresh media not cultured with cells and a blank buffer as controls. Based on MALS and AFM particle sizing, no particulate materials within the size range of sEVS were detectable in these controls.
3. Consistent cell seeding protocols and cell viability tests were used across multiple EV isolation runs to ensure the reproducibility and quality of the isolated sEVs. All chemicals, buffers and other reagents used were molecular grades.

Additional References:

1. Théry, C. et al., Minimal Information for Studies of Extracellular Vesicles 2018 (MISEV2018): A Position Statement of the International Society for Extracellular Vesicles and Update of the MISEV2014 Guidelines. *Journal of Extracellular Vesicles* **2018**, *7* (1), 1535750. https://doi.org/10.1080/20013078.2018.1535750.
2. Kornilov, R. et al., Efficient ultrafiltration-based protocol to deplete extracellular vesicles from fetal bovine serum. *J. Extracell. Vesicles* **2018**, 7.
3. Thierry, C.; Amigorena, S.; Raposo, G.; Clayton, A. Isolation and Characterization of Exosomes from Cell Culture Supernatants. *Curr. Protoc. Cell Biol.* **2006**, 3, 1–29.

The sEV samples were vitrified to preserve the native structure of the samples. Freshly prepared samples were plunge frozen in liquid propane using a Vitrobot (FEI). Cryo-EM was performed on TF20 High-Resolution CryoEM (FEI). The tetraspanin assay, utilizing common sEV surface marker antibodies immobilized on substrates- CD81, CD63 and CD9 was used to confirm the isolation of sEVs obtained from breast cancer cells.

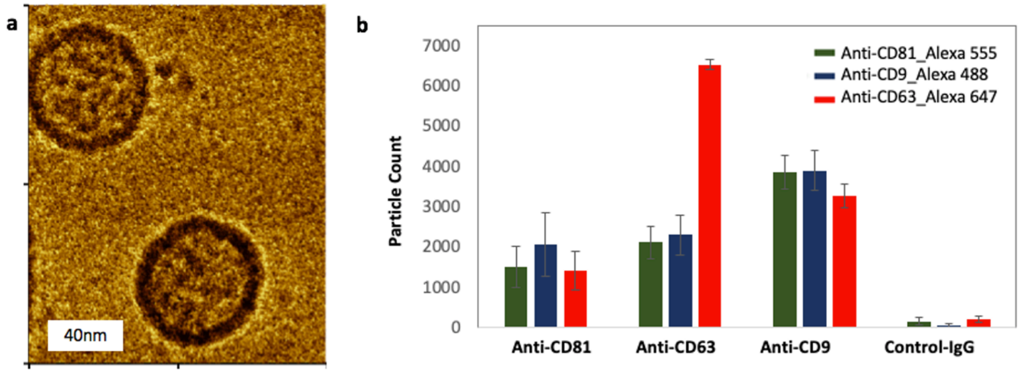

Figure S1. (a) The cryo-EM reveals lipid bilayer membrane enclosed vesicle structure of isolated sEVs (b) Graph shows particle counts (sEVs) bound via various validated capture antibodies using ExoView technology (NanoView Biosciences). Error bars show standard deviations of mean particle counts.

S2. Mass Spectrometry Proteomic Analysis of sEV isolates: We employed a shotgun proteomics workflow in which isolated sEVs were lysed, digested by sequential addition of lys-C and trypsin proteases, fractionated online using reversed phase chromatography and analyzed by tandem MS on a Thermofisher Fusion Lumos mass spectrometer. Data analysis was conducted using an in-house Galaxy-based bioinformatics pipeline utilizing MS-GF+, percolator, and Fido for database searching and filtering (<1% FDR at both peptide and protein levels using decoy database approach). Our sEV isolates were highly-enriched for EV proteins (i.e. 203 proteins annotated with the GO term “extracellular exosome” corresponding to an enrichment p-value of 2.8E-89) as well as membrane proteins (i.e. 104 proteins annotated with GO term “membrane” corresponding to an enrichment p-value of 1.7E-33).

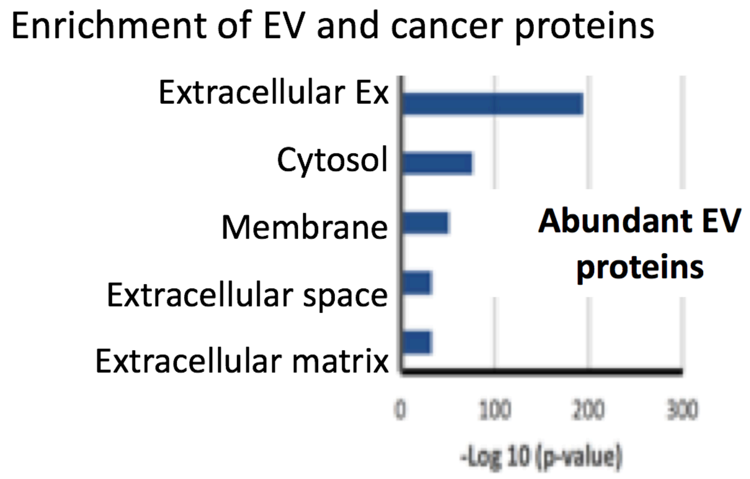

Figure S2 The breast cancer cell culture derived sEV isolates showed enrichment of sEV and cancer proteins in Mass Spectrometric analysis.

S3: dSTORM single-particle averaging: The spatial alignment and averaging of localization data from measurements of multiple structurally identical particles permitted a dataset to be obtained with much increased signal-to-noise ratio. Particle averaging analysis was applied to super-resolution image analysis. Reconstruction and data processing of recorded sEV images was performed using Fiji and QuickPALM software. The *d*STORM images were reconstructed with a pixel size of 10 nm. Output text files containing a list of the x and y coordinates and the precision of all detected molecules in the time-series, were used to further assess variations in the average particle sizes between sEV samples. To increase the efficiency and accuracy of our dSTORM analysis, we performed a quality check to discard all signals not corresponding to individual nanoparticles. We excluded very small objects (likely single fluorescent or soluble lipid molecules) and very big objects (likely aggregates of multiple nanoparticles), and high aspect ratio objects (e.g., dimers of nanoparticles, microvesicles).

Additional References:

1. Sigal, Y. M., Zhou, R. & Zhuang, X. Visualizing and discovering cellular structures with super-resolution microscopy. *Science* 361, 880–887 (2018).
2. Henriques, R. et al. QuickPALM: 3D real-time photoactivation nanoscopy image processing in ImageJ. *Nature Methods* 7, 339–340 (2010).

S4: STED imaging: A Leica TCS SP5 STED confocal system (Leica Microsystems, Wetzlar, Germany), equipped with a 640-nm pulsed diode laser (PicoQuant, Berlin, Germany) was used for excitation combined with a pulsed ultrafast Ti:sapphire infrared laser (Mai Tai Broadband; Spectra-Physics, Santa Clara, California), fully tunable from 710 nm to 990 nm. The sEVs were fixed and CD63 stained with Abberior STAR 635 anti- CD63 (Abberior® Dyes | Sigma-Aldrich). Images were filtered and flattened using SPIP 5.8 Image Metrology, Horsholm, Denmark. STED imaging was used as a comparative technique in parallel to the dSTORM analysis.

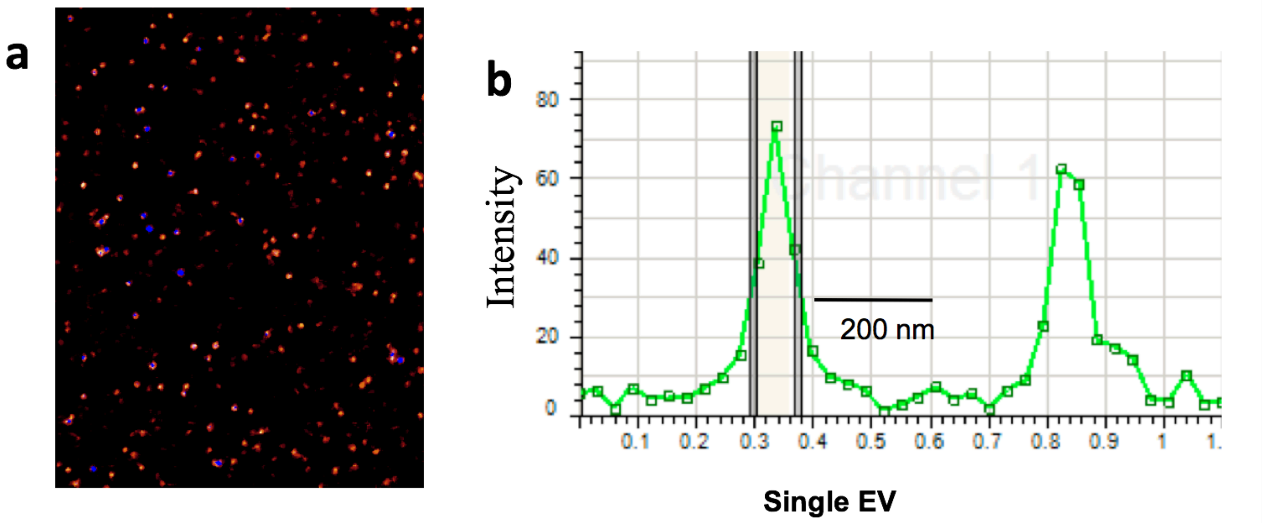

Figure S3 The dSTORM measurements on sEV isolates were validated using STED imaging in parallel. (a) STED imaging of sEV isolates (b) Representative line profile indicating the resolution of STED imaging enabling the resolution of single particles in sEV isolates.

S5: Biophysical characteristics of sEV particles obtained from MCF-10A breast cancer cells. Compared to MCF-7 and MDA-MB-231 small EVs, the measured size for MCF-10A derived sEVs were larger, and roughly two-folds less abundant based on AFM and MALS data (Table s1).

Table s1. Biophysical characteristics of sEV particles obtained from MCF-10A breast cancer cells.

		**AFM**		**MALS**
**Isolation Method**	**Cell line**	**Particle counts**	**Mean ± St dev. Particle diameter (nm)**	**Median Particle diameter (nm)**
**UC**	MCF-10A	325	80.0 ± 8.3	78.0
**UCg**	MCF-10A	180	77.0 ± 1.6	74.2

S6: AFM topographic scans show differences in residual particulates among blank controls from polymeric (PT) solution and immune-affinity (IA) beads method.

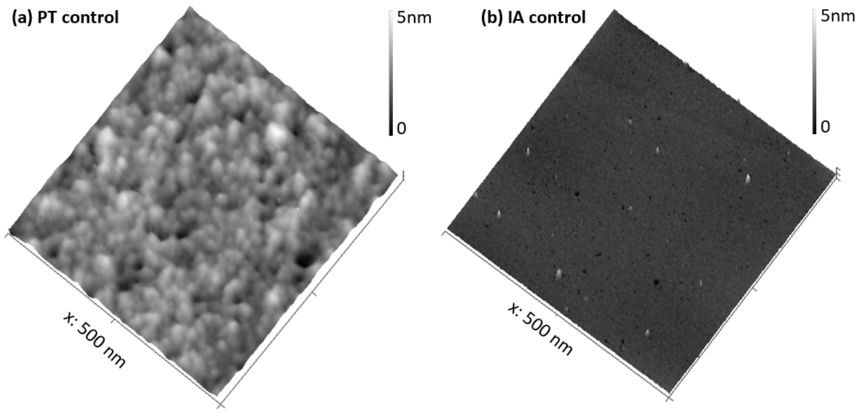

Figure S4. Differences in residual in residual particulates among blank controls from polymeric (PT) solution and immune-affinity (IA) beads sEV isolation methods.

S7: Roughness profiles of single EVs.

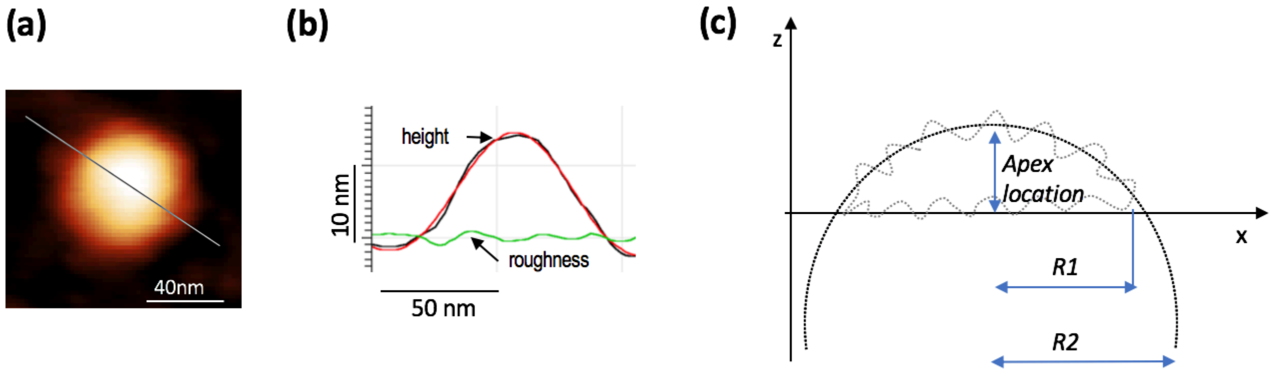

Figure S5. AFM based roughness analysis of single sEVs (a) AFM topographic image of single sEV and (b) cross section profiles. A quantitative surface roughness (green line) analysis of EVs involves subtracting the surface topography from the spherical fit. (c) A two-dimensional schematic illustration of a non- linear least squares fit of an AFM EV particle topography to a spherical shape showing radius of the sphere fit (R2), the apex, and radius of the particle (R1) at z=0, after reduction of the substrate background roughness.

Additional References:

1. Woo, J.; Sharma, S.; Gimzewski, J. The Role of Isolation Methods on a Nanoscale Surface Structure and Its Effect on the Size of Exosomes. *J Circ Biomark* **2016**, *5*. https://doi.org/10.5772/64148.
2. Hsu, C.-P.; Ramakrishna, S. N.; Zanini, M.; Spencer, N. D.; Isa, L. Roughness-Dependent Tribology Effects on Discontinuous Shear Thickening. *PNAS* **2018**, *115* (20), 5117–5122. https://doi.org/10.1073/pnas.1801066115.
